# Lessening barriers to healthcare in rural Ghana: providers and users’ perspectives on the role of mHealth technology. A qualitative exploration

**DOI:** 10.1186/s12911-020-1040-4

**Published:** 2020-02-10

**Authors:** Prince Peprah, Emmanuel Mawuli Abalo, Williams Agyemang-Duah, Hayford Isaac Budu, Emmanuel Appiah-Brempong, Anthony Kwame Morgan, Adjei Gyimah Akwasi

**Affiliations:** 1 Department of Geography and Rural Development, Kwame Nkrumah University of Science and Technology, Kumasi, Ghana; 2 Department of Nursing, Kwame Nkrumah University of Science and Technology, Kumasi, Ghana; 3 Department of Health Promotion and Disability Studies, Kwame Nkrumah University of Science and Technology, Kumasi, Ghana

**Keywords:** mHealth, Healthcare, Barriers, Users, Rural Ghana

## Abstract

**Background:**

Key barriers to healthcare use in rural Ghana include those of economic, social, cultural and institutional. Amid this, though rarely recognised in Ghanaian healthcare settings, mHealth technology has emerged as a viable tool for lessening most healthcare barriers in rural areas due to the high mobile phone penetration and possession rate. This qualitative study provides an exploratory assessment of the role of mHealth in reducing healthcare barriers in rural areas from the perspective of healthcare users and providers.

**Method:**

Semi-structured interviews were conducted with 30 conveniently selected healthcare users and 15 purposively selected healthcare providers within the Birim South District in the Eastern Region of Ghana between June 2017 and April 2018. Data were thematically analysed and normative standpoints of participants were presented as quotations.

**Results:**

The main findings were that all the healthcare users had functioning mobile phones, however, their knowledge and awareness about mHealth was low. Meanwhile, rural health care users and providers were willing to use mHealth services involving phone call in the future as they perceived the technology to play an important role in lessening healthcare barriers. Nevertheless, factors such as illiteracy, language barrier, trust, quality of care, and mobile network connectivity were perceived as barriers associated with using mHealth in rural Ghana.

**Conclusion:**

The support for mHealth service is an opportunity for the development of synergistic relationship between health policy planners and mobile network companies in Ghana to design efficient communication and connectivity networks, accessible, localised, user-friendly and cost-effective mobile phone-based health programmes to assist in reducing healthcare barriers in rural Ghana.

## Background

The topic of removing barriers to healthcare services to improve access is a noteworthy matter in Ghana, especially in rural and remote areas [1, 2]. Over the years, Ghana's health system has been characterised by multifaceted access issues despite recent gains [2]. Certain factors such as inadequate professional staff, inadequate basic equipment, infrastructural deficit [2, 3], poverty [1–3], societal cultural norms and practices, distance [2, 3], transportation and lack of health insurance [2, 3] have acted severely as barriers to formal healthcare use in most parts of Ghana, particularly the rural areas [1]. However, innovative solutions to these multifaceted barriers are emerging recently [4]. Currently, the health sector of Ghana appears to be embracing new technologies in service delivery considering the ongoing policy of using drones to supply essential drugs and medical equipment to health centres, especially in rural and remote areas [5]. This technological direction is considered to fit perfectly into government’s plan of achieving access to universal healthcare in the country [5].

One technology that has also emerged and is heavilly utilised especially in developed countries is mHealth [4, 6]. This innovation involves using mobile phones to access formal healthcare from remote areas either through instant messaging for treatment observance, meetings over cell phones, interactive voice response (IVR) frameworks, and video conferencing between patients (healthcare users) and providers including medical doctors and nurses [6, 7]. This technology is proving effective by intensifying access to basic healthcare and health education not only in the developed world, but also in developing countries where several barriers exist in the health sector [8–11]. This is because mHealth is considered cost-effective as it removes the task of travelling from distant places to hospitals and health centres, especially in rural areas where health facilities are mostly located in large towns [1, 2, 12].

However, despite the high proliferation and ownership of mobile phones in rural Ghana, mobile phones seem not to have been better utilised in Ghana’s healthcare system. Again, mHealth has not been discussed within Ghana’s healthcare policy initiatives. This, therefore, serves as a motivation for this qualitative study to explore from the perspectives of both healthcare users and providers, the role of mobile phones in reducing barriers to healthcare in rural Ghana. The central questions asked by this study included: would patients feel comfortable having a consultation with a healthcare provider over the phone? would patients want a self-care intervention to be delivered via phones? would health care providers be willing to provide care to patients over the phone?

Knowledge base concerning providers’ and users’ attitudes and perceptions about mHealth is critical in ongoing technological directions in Ghana’s healthcare. The authors argue that knowing users’ and providers’ beliefs about the technology, is key in determining the best way for stakeholders within and outside Ghana’s health sector to plan for mHealth interventions in rural Ghana.

## Methods

### Study design and context

Considering the exploratory nature of the study, a qualitative approach was adopted to present a deeper understanding of mHealth’s role in reducing barriers to healthcare from the perspectives of users and providers in some selected health centres in the rural communities of Bunso, Nsuansa, Awisa, and Asawase in the Birim South District in Ghana. The district is covered by mobile networks operated by major telecommunications in the country. The qualitative approach offered a maximum interaction between the researchers and the interviewees which generated a meaningful collaborative effect [13]. As a result, the researchers and participants were interdependent and mutually interactive and remained open to new knowledge throughout the study.

### Participants and sampling procedure

The participants of the study were purposively selected formal healthcare users (30) and providers (15). The aim of selecting participants purposively was to obtain high-quality range of opinions and views regarding the role of mHealth in lessening barriers to healthcare in rural Ghana. To recruit the users, potential participants were selected from the general adult population including opinion and community leaders. Initial briefing of the overall objective of the study was given to potential participants and those who were interested in participating were given further details and included in the study. To recruit the providers (nurses and medical assistants), emails containing an introductory letter with a fax-back reply were sent out to all the healthcare providers at the health facilities of Bunso, Nsuansa, Awisa, and Asawase with ten agreeing to participate while a follow-up of telephone calls led to the recruitment of five further providers. Aside from this, to be included in the study, participants should be 18 years or above (constitutionally matured age in Ghana) and own a mobile phone.

### Data generation tool and procedure

Two different interview guides were developed for the users and providers respectively. The users’ interview guide was translated into Twi, the local dialect of the study area given the difficulty in expressing themselves fluently in the English language. Although the questions for each group focused mainly on the broad aims of the study, they slightly addressed different aspects of the issue. The development of the guides was informed by relevant existing qualitative literature on mHealth [14, 15]. The guides were also field tested with two participants (one user and one provider) who were outside the study sample but from the study site. Overall, the field testing informed the researchers of some necessary minor modifications especially in the instrument format, sequence and concepts. After the changes from the field test, the final guides focused on views, experiences, and perceptions regarding mHealth’s role in reducing healthcare barriers in the rural areas. It also covered perceived mHealth benefits and challenges as well as preferred mHealth services. In all, 45 in-depth interviews were conducted comprising 15 and 30 interviews with providers and users respectively between June 2017 and April 2018. The users included the general public, community leaders, teachers and other civil servants whilst the providers were nurses and medical assistants. The interviews were conducted by the first author who has a social science background as well as a native to the study region and for that matter speaks the local dialect and understands the local cultural setting. The opening question asked participants to give an account of their socio-economic backgrounds and ownership of mobile phones. Participants were also asked to provide details of their experiences with regard to knowledge and use of mobile phone for healthcare. More importantly, participants were asked to offer opinions on mHealth benefits in relation to rural healthcare: how they perceive mHealth to reduce barriers to healthcare in rural areas. These questions generated further arguments and discussions which yielded in-depth data for the study. The final question offered the participants the opportunity to describe possible challenges associated with mHealth implementation in rural areas and their willingness to adopt and use a mobile phone for healthcare purposes. Interviews were conducted in both Twi with most of the users and English with the providers. With prior consent from the participants, all the interviews were audio-recorded, and each interview session approximately ranged between 30 and 60 min. For flexibility purposes, the guides were utilised in response to how the participants responded during the interviews. Semi structured interviews were selected for this qualitative study as the method provides insight into the perceptions and behaviours of participants, which is embedded in their social and cultural contexts [16, 17].

### Data analysis

Data for the study were analysed thematically involving several steps [18]. After the data collection, all non-English recorded data were transcribed verbatim and translated by all researchers independently into English. The transcripts were read and re-read by the researchers for data familiarisation in order to gain an in-depth feeling as well as getting a better meaning of the participants’ words. We initially conducted open coding of the data, followed by a selective coding. These generated several themes after careful multiple readings of the transcripts. Finally, we performed a thematic analysis based on the data content. Themes were compared with the responses to identify common trends, similarities, and contrasts. The thematic data analysis offered the opportunity to identify, analyse and report patterns within data and helped to organise and describe the data in rich detail. We conducted full data verification where all the transcribed and coded data were checked through proofreading against the original audios and handwritten notes to ensure accurate and quality data for the study. The study results were presented under specific broad themes and key subjective views of the participants were presented using quotations.

### Trustworthiness

Trustworthiness in this study was enhanced by emphasising credibility, transferability, confirmability and dependability [19–22]. The summaries of the study results were shared among the study participants. The participants also affirmed that the findings reflected their expressed views, feelings, and experiences. The participants’ prolonged engagement with each interview session lasting approximately an hour was also key. There was external auditing and peer debriefing involving an outsider researcher with experience in qualitative research to examine the research process especially documents such as the transcripts, recorded interviews and handwritten notes to provide feedback to enhance accuracy and credibility. The researchers also reflected on their own biases and prejudices and bracketed and controlled them before the data were collected.

## Results

### Participants’ demographic descriptions

Regarding the users, 17 were males and 13 being females participated. The users were comparatively uneducated with an aggregate of 16 having no form of formal education and just 8 and 7 with secondary and primary education respectively. Pertaining to users’ age, the study found that mainstream of the participants was within the age category of 31–50 years. Most of the users were self-employed (24) and were engaged in informal economic ventures like conventional peasant farming, artisanal works, and petty trading, which was mirrored in the overall moderately low-income levels, and with the majority receiving monthly income less than GH¢250 ($56.95). Conversely, providers who partook in the study were largely females (10), with only 5 males. It can also be inferred that providers are exceedingly educated with all respondents attaining tertiary qualification and received average estimated monthly income ranging between GH¢ 1000–3000 (USD 227.27–681.82). Majority of the respondents were within the age group of 20–40 years. (See Table 1 for further details of the study participants’ background information).

**Table 1:** Participants’ background characteristics

Item	Users *N* = 30	Item	Providers *N* = 15
Gender		Gender	
Male	17	Male	10
Female	13	Female	5
Education			
None	16		
Primary	4		
Secondary	5		
Tertiary	5		
Age (years)		Age (years)	
Below 20	1	21–30	8
21–30	7	31–40	5
31–40	17	Above 40	2
Above 40	5		
Occupation		Occupation	
Self-employed	24	Nurses 10	
Institutionally employed	6	Medical Assistants 5	
Average Monthly Income (₵)		Average Monthly Income (₵)	
Less than 250	21	Less than 1000	1
More than 250	9	1000–2000	10
		Above 2000	4

### Summary of major themes and sub-themes

In all, eight and seven interlinking main and sub-themes were respectively identified based on the analysis of accounts of the sample participated in the study. The perspectives of the providers’ and users’ attitudes and perceptions of the role of mHealth in lessening barriers to healthcare in rural Ghana were the main constructed categories from the data. (see Table 2 for details).

**Table 2:** Key themes and associated sub-themes

Low mHealth knowledge and awareness among healthcare users	
High mHealth knowledge and awareness among healthcare providers	
Low mHealth utilisation among healthcare users	
High mHealth utilization among healthcare providers	
Perceived benefits of mHealth in relation to reducing barriers to healthcare in rural areas • *Reduce transportation problems* • *Reduce health care cost* • *Time saving* • *Reduce workload on professionals and pressure at health centres*	
Preferred mHealth service	
Willingness to adopt/use mHealth	
Perceived mHealth challenges • *Illiteracy and technological incompetency* • *Network failures* • *Other fears and concerns*	

### Low mHealth knowledge and awareness among healthcare users

Low mHealth knowledge and awareness were observed among the users. They were unaware that they could use their mobile phones to either text or call a healthcare provider for healthcare purposes:

*‘I do not know about mHealth and I have not heard about it before. We use our phones to make calls but we do not call doctors for health information even when we need it urgently, because we do not know’* [Female user].

Another participant also related that:

*‘We make calls and text messages but we do not know we could also call doctors for treatment on phone. We know that if you are sick you have to walk all your way to the health centre to see the doctor’* [Female user].

However, few users were privy to the use of mobile phones for healthcare services. They understood that primary healthcare and health education messages could be delivered through mobile phones. They were aware of some existing mHealth services available for healthcare users. They specifically mentioned text messages delivered from phone operators or mass media:

*‘I sometimes receive message on my mobile phone to send some short code to a particular number for health tips’* [Male user].

In relation to this, another user also noted that:

*‘With text messages, I regularly receive from the mobile network I use. They always send a message that I should text a message to a number for health information concerning my personal health’* [Male user].

### High mHealth knowledge and awareness among healthcare providers

Observations from providers’ participants highlighted an awareness of mHealth, with most having a clear/clearer understanding of the mHealth technology. Additionally, healthcare providers use mobile phones to access personal health information. However, the interaction between providers and users on the phone concerning healthcare issues seldom occurs. They maintained that most of the users are unaware of using their mobile phones to text or call a doctor for treatment or health information:

*‘For us, we regularly call or text our colleagues for health information or treatment on phone and it is a common practice among us. We also search for personal health information with the use of our phones’* [Male provider].

*‘Using a mobile phone for healthcare is part of the medical profession because we usually consult through phones. We the providers regularly seek healthcare information with the use of phones. However, the users hardly do it which I think it is because they are unware of such opportunity and even those who are aware hardly utilise it’* [Male provider].

### Low mHealth utilisation among healthcare users

Similar to the knowledge of mHealth, the study found that most of the user participants had not used mHealth services with few user participants using their phones to access healthcare either through calling or texting a provider for treatment or health information. However, some participants reported that they occasionally receive text messages from mobile network operators to send a code to a specific number for health information but they mostly decline doing so because they did not believe in the authenticity of the message as well as the quality of the resultant health information:

*‘I have never used my mobile phone to access healthcare before. In the first place, I do not know I could use my phone for healthcare. I occasionally receive text message to get health tips by sending some code to a number but I have never done it before’* [Female user].

Another user also emphasised with doubt that:

*‘Using my mobile phone for healthcare ? How is it possible my brother? I doubt it. In fact, I have never used my phone for healthcare before, the same to any member in this household. We all go to the health centre for treatment whenever we are not well’* [Male user].

The few user participants who had used their mobile phones for healthcare shared their experiences. They described mHealth as an easy and simple way through which they can contact healthcare providers, especially during an emergency and outside the usual health provider hours:

*‘I once used my mobile phone to seek for health information on a personal health issue. The information I got really helped me to treat that health issue which worried me for a long period of time. I think using a mobile phone for healthcare is simple and easy’* [Male user].

*‘Sitting at home and calling or texting a doctor for treatment is so simple and easy. Recently, I have begun to consult providers on phone without going to the health centre when I am not well and I think it is really helping. It is so simple and easy’* [Female user].

### High mHealth utilisation among healthcare providers

High prevalence of use of mHealth among the providers who participated in the study was observed with all the providers having used mHealth before. Provider participants explained that the use of mobile phones in health delivery has become part of their profession and cannot do away with the service of mHealth:

*‘I regularly use mobile phone for healthcare. I consult a colleague provider on phone for health information or treatment. Also, I occasionally seek health information online with my phone’* [Male provider].

*‘With this profession, mobile phones have become indispensable. We consult here and there with the use of mobile phones. When I need any health information I consult a colleague or search it online mostly with the use of phone’* [Female provider].

### Perceived benefits of mHealth in relation to reducing barriers to healthcare in rural areas

All the study participants exhibited good attitudes toward, and positive perceptions about, mHealth and showed strong interest in enhancing mHealth services in terms of its application by making the technology locally friendly and culturally sensitive. In most rural areas, such as the study prefecture where diverse barriers such as cost, transportation, limited healthcare centres and qualified medical staff, distance, among others exist, all the study participants saw mHealth as a viable technology for improving healthcare delivery. All the study participants perceived several advantages that are associated with mHealth which will help remove most healthcare barriers in rural areas:

#### Reduce transportation problems

All the study participants explained that using mobile phones for healthcare will reduce the cost involved in moving from a place of residence to health centres as most of the available health facilities within the district are inaccessible to them because of their locations and cost of transportation. Aside from the proximity of the health centres, which demands high transport cost, the poor conditions of road networks that connect them to the health centres were also highlighted. Thus, all the participants perceived mHealth as a solution to this transportation barrier:

*‘I think using mobile phone to call for help from a doctor will save us the cost involved in going to the health centre personally. You see, the health centres in this district are very far from us and it takes high cost to reach the centres. Moreover, I believe you have witnessed the kind of road network you used when coming? It is very bad. So, if I sit at home to call a doctor on phone to tell him/her about my problems for solution, I think all the transport problems will be reduced’* [Male user].

*‘I think it is a great idea for our healthcare users who reside in rural areas to use their mobile phones to access healthcare by way of either calling or texting for us to provide them with the needed help. It will help reduce their cost involved in getting to the health centre. Most of the users come from very remote areas and this takes a lot of money from them. Also, the road network is very terrible and most of them go through a lot of difficulties before they get here. So, I think the use of mobile phones to reach us for health assistance will help reduce both transport cost as well as the hustle they go through because of the bad roads’* [Female provider].

#### Reduce healthcare cost

All the study participants evaluated the cost of a phone consultation and cost of a face-to-face consultation and perceived mobile phone consultation to be cost-effective. One observation was that most of the user participants did not access formal healthcare and most of the people sought healthcare from informal healthcare providers, particularly due to the issue of the cost involved in accessing formal healthcare. As a result, both the users and the providers in this study perceived mHealth as a good remedy to this situation. They explained that using mobile phones for healthcare will eliminate face-to-face consultation costs such as card fees, possible hospital admission fees, among others:

*‘I do not like going to the health centre when I am sick because of money issues. When you go, they even ask for card fees and other charges. So, I think using mobile phones to call the nurses to prescribe medicine for you without you going to the health centre will reduce the cost of seeking health care from health centres. With this, I do not need to pay any card fees’* [Female user].

*‘One thing I have observed in this community is that most people do not come to the health centre because of financial issues. So, most of the people prefer going to local practitioners for treatment than the health centres. Some of the people do not have money for transport and also cannot afford the hospital charges. With mHealth, the person can sit at home and preferably use the transport cost to buy the prescribed drugs’* [Male provider].

#### Time-saving

Interestingly, all the study participants perceived mHealth as a time-saving technology in healthcare delivery and offered an explanation that using a mobile phone for healthcare will ease healthcare users from the time and hassle with travelling to access healthcare at health centres. They maintained that in most rural areas like where they reside, travelling to access healthcare at health centres which is predominantly located at the district capital and other bigger towns involves tremendous difficulties mostly because of bad roads. Aside from the travelling difficulties, all the participants also mentioned the long waiting hours and difficulties at the health centre and found mHealth as a good solution to these difficulties. Thus, they specifically saw using a mobile phone for healthcare as a time saving approach:

*‘To me, I think it will save time. The time for travelling and waiting hours at the health centre, all will be saved’* [Female user].

*‘I believe the use of mobile phones for healthcare will save our time and the users as well. The users can be saved with time to be here and waiting time as well. With mHealth, they can sit at home and access healthcare without wasting time to be here’* [Male provider].

#### Reduce workload on professionals and pressure at health centres

Providers perceived mHealth as a technology which could reduce their workload and reduce the pressure at health centres as more people would access healthcare via their mobile phones. Providers explained that a lack of qualified doctors and medical staff increased their work load, and that strain on their resources is higher in rural areas with limited health centres. They maintained that mHealth will expand access to healthcare in the rural areas since everyone who will call or text can be attended to against the situation where people come to the health centres and return unattended due to pressure:

*‘I think mHealth will reduce our workloads and pressure at the health centre. You see, we are very limited here serving a larger population. With mHealth, not everyone will prefer to come to the health centre, definitely some will choose to call for treatment and as a result, reduce pressure and workload. Also, with mHealth, I think access to healthcare will be expanded since anyone who calls will get treatment unlike the situation where people come and leave unattended to’* [Male provider].

### Preferred mHealth service

All the study participants, especially the users preferred mHealth services involving self-care intervention such as direct calling of healthcare provider for medication and health information. Few of the participants also preferred messaging. The study participants explained that these mHealth services would be simple, easy, and user friendly. In addition, most of the participants described the mHealth service of calling and messaging as less expensive:

*‘I like the calling because it will be easy and simple. All that I will need is the number of the provider. Dialing the number would not be difficult for me as messaging because I cannot read. With calling, I can write the number somewhere so anytime I need help then I call the doctor’* [Female user].

*‘We like the calling because it is simple and easy. For calls, we all make calls every day so we can do it but for other services such as messaging most of us cannot do because we cannot write and read’* [Male user].

### Willingness to adopt/use mHealth

Interestingly, all the study participants showed strong interest in using mHealth services in the future, with users specifically explaining that using a mobile phone for healthcare has the ability to bring them closer to healthcare providers considering their remoteness from the providers. However, the users recommended fixed lines or call centres where their calls can be given quick response. On the other hand, the providers expressed that the use of mobile phones for healthcare will also enhance their service delivery at the rural areas and expand healthcare access. Moreover, all providers emphasized the efficacy of mobile phones to reduce the issues of cost and transportation, which are the most pressing barriers to formal healthcare in rural areas:

*‘I am ever ready and willing to use mHealth. I think it is a good technology which will help us to have access to healthcare in this community. When we use this, all the transportation cost and problems will be reduced. I am looking forward for that’* [Male user].

*‘For us in the health sector, we use mHealth a lot. However, we hardly use it among the users in this community. I think it is high time we started using it with the users. When it is done that way, various forms of healthcare barriers will be lessened. We are all for mHealth’* [Male provider].

### Perceived mHealth challenges

The study participants highlighted some issues they perceived will be a challenge to the use of their mobile phones for healthcare in rural areas. Issues such as illiteracy and technological incompetency, network failures, among other fears and concerns were broadly mentioned.

#### Illiteracy and technological incompetency

The study found that most of the users were unable to effectively use their mobile phones due to their inability to read and write and as a result expressed concerns about the effective use of mHealth services which involves reading and writing. Some of the users specifically mentioned that they always delete text messages that they receive without reading them because they could not read and understand them:

*‘I think the writing, sending and reading of messages will be our problem. Most of the people here do not know how to send text messages, especially we the adults. Personally, I do not know how messaging is done’* [Female user].

*‘With mHealth service which involves reading, it will be very difficult for some of us to use. This is because we cannot read nor write, so we will hardly get the message that will be sent to us. I always delete any message I receive as soon as it comes because I cannot read’* [Male user].

#### Network failures

All the study participants echoed the issue of network unreliability by describing the network at most rural areas including the study area as very bad and unreliable. As a result, calling or texting a provider for healthcare may delay because of the poor network:

*‘The network in this community is very poor. When you want to call someone, it will take you a number of hours or days to get network to make the call. We have some identified place where the network is somehow good that we stand there always to make calls. But when you move from that place you will go out of reach and I think that would not be good for mHealth’* [Male user].

*‘One challenge I think will work against mobile phone usage for accessing health care among rural dwellers is poor and unreliable mobile network at their residence. Most of the people live in areas which are very difficult to reach them on phone and I think that will be a challenge when using mHealth’* [Female provider].

#### Other fears and concerns

On the part of the users, concerns such as language barrier, quality of service, reliability, and trust were broadly highlighted. Most of the users mentioned language as a possible challenge to mHealth. They emphasised that some of the professionals who they will talk to on phone might not be able to understand the local dialect (Twi) which will make it difficult for them to communicate effectively with them as these professionals mostly speak the English language which they do not comprehend when communicating with them. Thus, the users preferred local language for the communication. Aside the issue of language, a lack of trust in mHealth services was reported by some of the users largely because they will not be able to see the person they will be communicating with and therefore fear their calls may not be received by qualified medical professionals:

*‘My issue has to do with language. Is the person who will communicate with us going to speak our local language? If not then how would the person understand us and vice versa? It will work best if local language is used for the communication between us and the providers’* [Male user].

*‘My concern is how would I know that I am speaking to the right person since I cannot see the doctor?’* [Male user].

On the other hand, the providers expressed their concerns about prescribed medicine, since they believe most patients from rural areas are not being able to understand the name of the drugs they need to buy and prescribed medicine sometimes not being available in remote areas:

*‘One concern is about how the users can get the prescribed drugs correctly and use them as indicated. One thing is that the drugs that we may prescribe on phone for the patients may not be available in the rural area’* [Female provider].

## Discussion

This qualitative study assessed the role of mobile phone technologies in reducing healthcare barriers in rural Ghanaian context. In this context, the present study is novel, highly engaging and instigative, as it provides the first snapshot evidence on rural healthcare users’ and providers’ experiences, attitudes and perceptions about mHealth. The study findings fit into the ongoing policy initiatives and technological directions of Ghana’s healthcare sector. For instance, the use of drones to deliver medicine, blood and other critical healthcare equipment involve communication through mobile phones.

The study observed low knowledge and awareness of mHealth among the healthcare users. This poor knowledge of mHealth has been reported previously by other studies elsewhere [4, 14, 23–25]. By implication, rural healthcare users use mobile phones for many purposes but may not be aware of the use of the technology for medical purpose. Such lack of knowledge about the existence of mHealth is not uncommon in remote areas in developing countries such as the study areas where education and capacities to read, write and operate mobile phones efficiently are low [26–29]. However, for a maximum utilisation of the potential benefits associated with mobile phone-based medical intervention in the future, poor mHealth knowledge and awareness need to be addressed through rapid mHealth awareness creation and public education programmes.

On the contrary, the study found that mobile phone utilisation for healthcare was uncommon among the healthcare users. Fortunately, healthcare providers in this study found mHealth to be part of the medical profession as regular consultation and discussion about healthcare take place among themselves and patients in some occasions. With this finding, implementing any phone-based healthcare intervention may not come with a resistance from the part of the providers since they are into the practice of the use of phones for healthcare and information. However, on the part of the users, the study discovered that their non-utilisation of mHealth was largely as a result of their socio-demographic characteristics, particularly literacy levels. Other researchers working in low literacy populations have reported similar problems in accessing technology-based services [6]. As observed, the poor education levels among the user participants prevent them from using their mobile phones for healthcare. Most of the users’ inability to read and write make them handicapped in using their mobile phones for healthcare. For example, they usually do not read text messages or act upon messages received as they are unable to operate their phone options or understand the message itself. This finding agrees with Khatun et al.’s [14] evidence that most rural healthcare users did not use mHealth because half of them had no knowledge about the use of text messages. This low knowledge about mobile phone application among most rural dwellers is considered a barrier to mobile phone-based healthcare intervention and calls for special capacity building and training for users of technology-based systems in the future [14, 23, 30, 31]. The enhancement of people’s capacity in mobile phone operation will promote future mHealth service usage among rural dwellers.

Despite the low utilisation of mHealth technology among the user participants, this study provides useful information to suggest that stakeholders in the health arena, specifically providers and users, believe in the potential of mobile phone in reducing healthcare barriers in rural and remote areas due to the perceived benefits associated with the innovation. The participants evaluated the use of mobile phone for healthcare to be cost-effective, thus reducing costs and saving time. The removal of direct costs involved in accessing healthcare including consultation and transportation costs make mHealth a good technology for lessening barriers in rural areas. This evaluation of the benefits of mHealth in reducing healthcare barriers in rural areas was also reported by Khatun et al. [14] in rural Bangladesh where participants calculated mHealth to be an inexpensive way to consult a healthcare provider, as doctors’ consultation fees and other indirect costs (e.g. travel, wages) were not involved. The difficulties involved in reaching health centres in the study area due to poor transportation network and consultation time will be reduced with the use of mHealth since one need not to be at the health centre for treatment but can sit at home and access healthcare remotely. Providers perceived mobile phones-based healthcare intervention as an opportunity to reduce workload and pressures at rural healthcare centres. Due to the lack of qualified health professionals, especially in rural areas, providers believe that mobile phone-based healthcare intervention will ease their workload and increase access to healthcare in rural areas.

In agreement with Peprah et al’s [1] study in rural Ghana, the present study findings suggest that a mobile phone-based health care system can easily be adopted and implemented in rural Ghana, as all the participants expressed their willingness to adopt and use the intervention. The findings of the current study exhibit the practicability of using mobile phones to access health care at the rural areas of Ghana and other rural African communities. In rural societies with restricted accessibility to health services, mHealth connectedness could influence life and death resolutions in emergency circumstances [11, 24, 25]. Globally, mHealth works best when the users and providers are aware and informed about the intervention. This study provides evidence to suggest that both health care users and providers are enthusiastic supporters and campaigners of mHealth indicating easy mHealth integration into the national health care system.

Concurrent with Hounmanou et al. [24], participants in this study also preferred phone calls over other mHealth services due to the simple and user-friendly nature of phone calls. This finding contradicts previous studies which reported that SMS is the most preferred phone-based health care intervention method [14, 30, 32–34]. SMS messaging may not be appropriate in most rural areas of Ghana due to the illiteracy noted amongst users. The inability to read and write will significantly constrain and influence their choice of mHealth service, with phone calls providing a tool to overcome the difficulties inherent in illiteracy. However, Hounmanou et al. [24] suggested that IVR frameworks, with voice messages that can supplant mediations where text messages are necessary, have been implemented in Bangladesh and other nations [14] successfully indicating a possible successful implementation in other developing countries such as Ghana.

The present study demonstrated certain key issues that all the participants perceived to be challenges that are associated with mHealth implementation in the rural areas. On the part of the users, four main concerns were highlighted including socioeconomic factors, particularly illiteracy and language, mobile network problems, trust and reliability, and quality of care which support other previous studies [11, 23, 25]. However, providers’ main concern was that, patients may not be able to acquire the correct and right prescribed drugs at their place of residence since most drugs may not be available in the rural and remote areas. Illiteracy was also a key issue in this study since an effective mHealth technology operation requires adequate knowledge in mobile operation as well as the understanding of English language. This implies that, users must be literate in English. Language barrier has been identified as one of the drawbacks in the Argentinian text messaging programme to provide pregnancy and postpartum support to women [35] and India’s mHealth programme targeting tuberculosis patients [36]. Due to the educational background of the rural dwellers, the mode of mHealth programme delivery should be user-friendly and easy. Such mobile phone-based healthcare interventions should be considered designing them in local languages and train pilot populations on the use of the system for successful application. Another issue was trust and concerns about the quality of care. The user participants expressed concerns about the quality of healthcare providers in mHealth services. Participants raised issues about the patient’s inability to see the doctor over the phone and the likelihood of having no relationship with the providers. It has been found by previous studies that consumer insights of quality of service are a robust influencer of the utilisation of mHealth platforms [36]. Also, literature concerning trust has discovered that individuals have more trust in personal relationships and individual encounters with a service provider and less trust in provenance of information with which they have no close involvement [37]. This finding demands conscious efforts to create a healthier connection between the healthcare providers and users in the future for related projects to be successfully provided in Ghana. Khatun et al. [14] suggested that regular appraisals of the mHealth programme and Information Communication Technology policy guidelines will guarantee quality services and answerability to play a role in gaining their conviction for mHealth services. Moreover, by developing practice procedures to build up and keep up distant accessibility, these worries could be dispensed with. The advantages obtained from this kind of system administration could energise the formation of a joint agenda, innovative and profitable joint effort, and related positive health-related results in rural Ghana.

### The study strengths and limitations

Some strengths and restrictions of this study ought to be acknowledged to enable readers to put the interpretation of the present study discoveries in its context. To the best of our knowledge, this is the first qualitative study in Ghana that has provided a unique and detailed insight into the role of mHealth in reducing healthcare barriers in rural areas from the viewpoints of healthcare users and providers. Thus, the study exhibits a depth of understanding from the interpretations of beneficiaries of mHealth use (providers and users) and offers an imperative support to address the prevailing gap in knowledge. It also investigates Ghana’s health strategy agenda on a potential integration of mHealth services into the nationwide healthcare system. However, the study has some limitations which are premised on its methods particularly sample size and sampling procedures. Two important caveats must be added here: Because of the purely qualitative nature of the research that seeks to identify themes that are contextual and therefore cannot be assumed to be independent of the context and individuals, our results should not be regarded as representative to all the general population in Ghana. However, readers can relate the study findings to their settings based on local knowledge. Again, the study findings were based on responses from small purposively selected formal healthcare users and providers in rural communities. To this, a larger quantitative study would be required. The authors believe that these limitations are far outmatched by the benefits offered by conducting this first empirical study on mHealth role in reducing healthcare barriers in rural Ghana.

## Conclusion

This qualitative study suggests insights into the role of mHealth in reducing healthcare barriers in rural areas from the perspectives of healthcare users and providers in rural Ghana. The main findings were that healthcare users had low knowledge and awareness about the use of mobile phone for healthcare and as a result hardly use their phones for medical purposes. The study also provided evidence to suggest that rural healthcare users and providers are willing to use mHealth services involving phone call in the future as they perceived the technology to play an important role in reducing healthcare barriers through transportation and direct costs reduction, travelling hassles reduction and time savings.

With these findings in mind, mHealth service is an opportunity for health policy stakeholders and planners together with mobile operability networks in Ghana to design more accessible, localised and cost-effective mobile phone-based health programmes and policies to assist in reducing healthcare barriers while expanding access in the rural and remote areas. Importantly, it must be re-emphasised that whatever the issues associated with mHealth, the technology presents clear opportunities in terms of healthcare provision, which may bring benefits on the assumption that a variety of other conditions are in place to cater for the possible drawbacks.

## Data Availability

The datasets used and/or analysed during the current study are available from the corresponding author on reasonable request.

## Abbreviations

IVR: Interactive Voice Response
mHealth: Mobile Health
SMS: Short Message Service
